# G-CSF Prevents the Progression of Structural Disintegration of White Matter Tracts in Amyotrophic Lateral Sclerosis: A Pilot Trial

**DOI:** 10.1371/journal.pone.0017770

**Published:** 2011-03-14

**Authors:** Thomas Duning, Hagen Schiffbauer, Tobias Warnecke, Siawoosh Mohammadi, Agnes Floel, Katja Kolpatzik, Harald Kugel, Armin Schneider, Stefan Knecht, Michael Deppe, Wolf Rüdiger Schäbitz

**Affiliations:** 1 Department of Neurology, University Hospital Muenster, Muenster, Germany; 2 Department of Clinical Radiology, University Hospital Muenster, Muenster, Germany; 3 Department of Neurology, Charité - Universitaetsmedizin Berlin, Berlin, Germany; 4 Center for Stroke Research Berlin, Charité - Universitaetsmedizin Berlin, Berlin, Germany; 5 Cluster of Excellence NeuroCure, Charité - Universitaetsmedizin Berlin, Berlin, Germany; 6 SYGNIS Bioscience, Heidelberg, Germany; 7 Department of Neurology, EVK Bielefeld, Bielefeld, Germany; Julius-Maximilians-Universität Würzburg, Germany

## Abstract

**Background:**

The hematopoietic protein Granulocyte-colony stimulating factor (G-CSF) has neuroprotective and -regenerative properties. The G-CSF receptor is expressed by motoneurons, and G-CSF protects cultured motoneuronal cells from apoptosis. It therefore appears as an attractive and feasible drug candidate for the treatment of amyotrophic lateral sclerosis (ALS). The current pilot study was performed to determine whether treatment with G-CSF in ALS patients is feasible.

**Methods:**

Ten patients with definite ALS were entered into a double-blind, placebo-controlled, randomized trial. Patients received either 10 µg/kg BW G-CSF or placebo subcutaneously for the first 10 days and from day 20 to 25 of the study. Clinical outcome was assessed by changes in the ALS functional rating scale (ALSFRS), a comprehensive neuropsychological test battery, and by examining hand activities of daily living over the course of the study (100 days). The total number of adverse events (AE) and treatment-related AEs, discontinuation due to treatment-related AEs, laboratory parameters including leukocyte, erythrocyte, and platelet count, as well as vital signs were examined as safety endpoints.

Furthermore, we explored potential effects of G-CSF on structural cerebral abnormalities on the basis of voxel-wise statistics of Diffusion Tensor Imaging (DTI), brain volumetry, and voxel-based morphometry.

**Results:**

Treatment was well-tolerated. No significant differences were found between groups in clinical tests and brain volumetry from baseline to day 100. However, DTI analysis revealed significant reductions of fractional anisotropy (FA) encompassing diffuse areas of the brain when patients were compared to controls. On longitudinal analysis, the placebo group showed significant greater and more widespread decline in FA than the ALS patients treated with G-CSF.

**Conclusions:**

Subcutaneous G-CSF treatment in ALS patients appears as feasible approach. Although exploratory analysis of clinical data showed no significant effect, DTI measurements suggest that the widespread and progressive microstructural neural damage in ALS can be modulated by G-CSF treatment. These findings may carry significant implications for further clinical trials on ALS using growth factors.

**Trial Registration:**

ClinicalTrials.gov NCT00298597

## Introduction

Amyotrophic lateral sclerosis (ALS) is a devastating and incurable neurodegenerative disease with progressive loss of motor neurons. It is characterized by motor weakness and muscle wasting finally leading to death due to respiratory failure within 2–5 years after diagnosis [1]. A causal treatment for ALS is currently not available. The only approved pharmacological treatment opportunity is a continuous treatment with the NMDA antagonist riluzole. Riluzole prolongs life by 2–3 months, but an improvement in neurological function is barely noticeable [2]. Therefore treatment alternatives are urgently needed. A number of different drugs were tested in clinical trials but none of them proofed to be effective. The failures have mainly been ascribed to the inability of candidate drugs to cross the blood-brain barrier (BBB), to problems of insufficient dosing or to intolerable side effects [3], [4].

Recent studies have uncovered the neuroprotective and regenerative properties of the haematopoietic protein granulocyte-colony stimulating factor (G-CSF) [5], [6]. A number of mechanisms of action in the CNS have been identified, the most relevant relating to neuroprotection, neuroplasticity, stem cell proliferation and differentiation. Thus, G-CSF could be a promising therapeutic candidate for the treatment of neurodegenerative diseases. Given that the exact disease-causing mechanism of ALS is still unknown, a general trophic support to motoneurons, e.g. by growth factors such as G-CSF, could be a rational approach. G-CSF crosses the intact BBB, thus allowing an efficient peripheral delivery [6]. Moreover, G-CSF is in clinical routine for the treatment of haematological disorders for more than a decade and its pharmacological behavior and safety profile are well understood.

The purpose of this pilot study was to assess whether G-CSF treatment in ALS patients is feasible, and to explore potential subclinical effects of G-CSF on structural cerebral abnormalities by using a combination of voxel-based morphometry (VBM) and whole-brain, voxel-based diffusion tensor imaging (DTI) analysis.

## Methods

### Participants and Procedures

**Figure 1:**
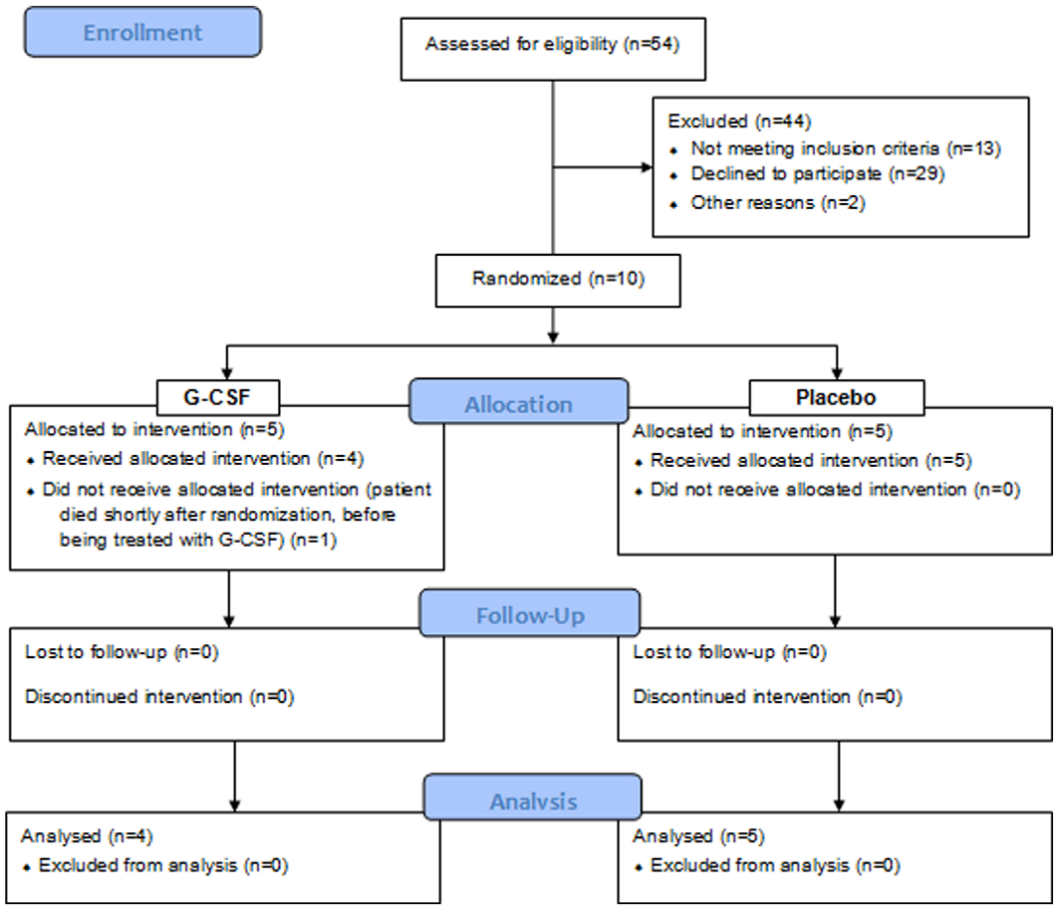
Consort Flowchart.

Ten patients (median age 58 years; range 45–71 years; 6 men, 4 women) with definite ALS based on revised El Escorial criteria were recruited for this double-blind, placebo-controlled and randomized study [7]. The CONSORT flowchart is shown as Figure 1. The here reported study, conducted according to ICH GCP guidelines, was originally planned as a larger arm in a registered recovery trial (ClinicalTrials.gov NCT00298597), but due to problems in patient recruitment was conducted and reported independently with a smaller number of patients. A group of 32 healthy subjects (17 women, median age 54.1 years, range 46 to 64 years) served as a control group for the cross-sectional image analyses. Patients taking riluzole were included if they were on a stable dose for at least 30 days before enrolment (for further details on patients, see Table 1). After enrolment, patients were randomly assigned to subcutanteous injections of G-CSF or saline solution (10 µg/KG/day G-CSF or 0.1 mL/KG/day placebo) during the first 10 days and from day 20 to 25 of the study. The study drug was given in a double-blind way. Study medication, and randomization to either G-CSF or placebo, was provided by the pharmacy of the University of Mainz. Filgrastim (recombinant human G-CSF produced in E. coli, solubilised in a buffer containing 10 mM acetic acid, 5% (m/v) sorbitol, 0.004% Tween 80, ph adjusted to 4.0 with NAOH), or saline as placebo were filled in identical-looking glass vials to ensure blinding. Vital signs and laboratory parameters were determined repeatedly throughout the course of the study.

**Table 1:** Patient demographics and clinical characteristics at baseline.

Parameter	G-CSF	Placebo
Age [years]	51.8±10.1	50.0±12.2
Gender (male/female)	1/4	3/2
Month since diagnosed	13.9±8.1	12.5±6.9
Duration of symptoms [month]	19.5±8.4	19.1±7.5
Years of education	11.9±3.1	12.1±2.3
ALSFRS at baseline	27.4±2.5	29.3±4.6
Total time for JTT at baseline [seconds]	52.4±11.3	54.6±8.1
Riluzole treatment since [month]	12.4±5.4	11.9±3.2
Onset of ALS-Symptoms	Bulbar: 0Lower limb: 2Upper limb: 3	Bulbar: 1Lower limb: 2Upper limb: 2
Blood pressure (systolic/diastolic) [mmHg] at baseline	134/79±13.5/11.7	137/83±14.2/10.7
heart rate [beats/minute] at baseline	78±13.4	73±10.9
body temperature [°C] at baseline	35.4±0.5	35.9±0.4

Differences were not significant for any parameter (all *P's*>0.05); mean ± SD given.

Each patient was physically examined and a questionnaire for the ALS functional rating scale (ALSFRS) was used to assess disease severity [8]. To assess motor hand functions and cognitive ability, the Jebsen Taylor Test (JTT) [9] and a comprehensive neuropsychological test battery were conducted, respectively. An experienced clinical neuropsychologist, who was unaware of the allocation of the patients, conducted the neuropsychological tests. Performances in five major areas of cognitive functioning were evaluated. Cognitive domains and their particular tests are listed in Table 2. The same test was not included in more than one cognitive domain score. Concerning the Rey Osterrieth Complex Figure Test, we also calculated the relative difference between both test results since results of delayed recall performance can be influenced by an impairment of initial copying. Due to the small number of subjects, we additionally compared mean results of each neuropsychological test with standardized normal values, adjusted for age, sex, and educational level (see Table 2 for baseline results). Differences were expressed semi quantitatively as normal, close below average, or far below average, respectively. A detailed description of each test can be found in Lezak et al. [10]. Additionally, safety endpoints were examined, that is, the total number of adverse events (AEs), the number of treatment-related AEs, and discontinuation due to treatment-related AEs. Secondly, laboratory parameters including leukocyte, erythrocyte, and platelet count, and vital signs (body temperature, blood pressure, heart rate) were assessed.

**Table 2:** Neuropsychological test results at baseline.

	ALS			Controls		
Cognitive domain and tests	Score (Percentile)	Evaluation	z-scores	Score (Percentile)	Evaluation	z-scores
**Dementia screening**			0.005			−0.002
MMSE	29.1	normal		28.9	normal	
Boston Naming Test	13.9	normal		14.0	normal	
**Attention and Working memory**			−0.032			−0.022
NAI- Digit Symbol Substitution	31.0 (56.2)	normal		33.1 (60.7)	normal	
- CWIT reading	56.8 (44.2)	normal		60.0 (50.4)	normal	
- CWIT colour naming	54.3 (29.0)	close below average		52.9 (27.9)	close below average	
WMS-R - Digit Span Forward	11.7 (51.8)	normal		12,6 (54.8)	normal	
- Digit Span Backward	11.7 (41.6)	normal		11.9 (42.0)	normal	
Trail-making test [A]	45.2 (33.4)	close below average		36.0 (50.1)	normal	
**Executive function**			−0.001			−0.011
CWIT - interference condition	17.7 (50.9)	normal		19.8 (52.8)	normal	
RWT - Letter fluency (‘S’)	16.2 (47.4)	normal		16.4 (49.0)	normal	
Trail-making test [B]	100.1 (49.8)	normal		96.0 (53.0)	normal	
**Visuospatial skills**			−0.081			0.045
ROCF - Copy	20.8			21.6		
- Delayed recall	12.4 (38.8)	normal		14.2 (42.0)	normal	
- Difference Copy- Delayed [%]	−29.3%			−29.9%		
**Verbal learning and memory**			0.027			−0.024
AVLT - Recall trial 1	6.8 (50.2)	normal		6.8 (51.3)	normal	
- Recall trial 5	12.2 (51.1)	normal		11.5 (30.5)	close below average	
- Total trials 1 to 5	48.2 (54.4)	normal		45.7 (49.0)	normal	
- Delayed recall	10.5 (55.8)	normal		11.3 (58.4)	normal	
- Recognition (True Positive – False Positives)	13.9 (59.5)	normal		12.8 (52.1)	normal	

Differences were not significant for any domain (all *P's*>0.05). SD  = Standard Deviation; NAI  =  Nuremberg Gerontopsychological Inventory; CWIT  = Color-Word- Interfer-ence Task; RWT  = Regensburg Word Fluency Test; WMS-R  = Wechsler Memory Scale-Revised; AVLT  = Auditory Verbal Learning Test [German Version]; RCFT =  Rey-Osterrieth Complex Figure; MMSE =  Mini-Mental State Examination.

### Image acquisition and analysis

Image data were obtained on a 3.0 T system with a high resolution structural T1-weighted 3D turbo-field-echo sequence (reconstructed after zero filling to 512×410×320 cubic voxels, edge length 0.5 mm), as well as T2-weighted, and FLAIR imaging. For DTI we employed echo planar imaging (EPI) with 20 diffusion directions (36 slices, thickness 3.6 mm, matrix 128×128, inplane resolution 1.8×1.8 mm).

MRI examinations of the ALS patients were performed at Day 0 and Day 100 of the study.

Diffusion tensor and FA (fractional anisotropy) field maps were calculated from spatially normalised images. The method was described in detail previously [11]. In brief, after correction for eddy currents with an in-house software, the EPI images were spatially normalized to the Montreal Neurological Institute (MNI) coordinate system following an optimized procedure. The diffusion tensor and FA field maps of all participants were calculated from the spatially normalized images. In a second step, all FA images were normalized to an FA template image also corresponding to the MNI coordinate space. Fiber direction maps were calculated on the basis of the largest eigenvector.

Patterns of cerebral atrophy were assessed using the automated and unbiased technique of VBM. An optimized method of VBM was applied using both customized templates and prior probability maps, implemented using SPM5. The processing steps were performed as previously described [12]. Briefly, all images were normalized to a customized template and segmented by the unified segmentation procedure in SPM5 using the customized tissue probability maps into gray matter, white matter, and CSF, followed by the hidden Markov random field clean-up step. All images were modulated, and smoothed with a 12-mm full-width at half maximum smoothing kernel.

Total brain tissue volumes, normalized for subject head size, were calculated from the high-resolution T1-weighted images, using the well-established cross-sectional version of the Structural Imaging Evaluation of Normalized Atrophy (SIENA) software (SIENAx) [13].

### Statistical Analysis

Data were testes for normal distribution with the Wilk-Shapiro test. Changes of ALSFRS, JTT and cognitive performance from baseline to Day 30 and 100 were examined between groups using a two sample t-test. Fisher's Exact Test was used to assess differences in the number of AEs between groups. Differences in laboratory parameters and vital signs were assessed with a two sample t-test.

Single neurocognitive test results were Z-transformed with a mean score of 0 and standard deviation of 1; mean Z-scores of cognitive domains were then calculated by taking the mean of the individual Z-scores. For timed tests, the sign of the Z-score was reversed so that improved performance resulted in a higher score in all tests. Differences in neurocognitive test results between groups were assessed with either two sample t- test or Mann-Whitney *U* test as appropriate. All data are given as means ± SD, unless stated otherwise. A two-tailed P value <0.05 was considered significant. All statistical analyses were performed using SPSS 16.

Voxel-wise statistical tests were performed on FA and gray matter values using Factorial ANOVA in SPM5 (http://www.fil.ion.ucl.ac.uk/spm/). We hypothesized that all ALS patients (at Visit 1) would show damage to white matter tracts reflected as decreased FA when compared to the 32 controls. This hypothesis was tested using cross-sectional comparisons, i.e. two-sample t-tests performed on Visit 1 data (pre-medication). Differences between both ALS-groups vs. healthy volunteers as well as each single ALS-group vs. controls were calculated. Secondly, differences in FA changes between ALS patients treated with G-CSF and the ALS-Placebo group in the 100 days following Visit 1 were assessed. This was tested using a factorial design, with VISIT (Visit 1, Visit 2) as the first factor, and GROUP (ALS-GCSF, ALS-Placebo), as the second factor. We hypothesized that there would be an overall group difference in FA change between these groups, manifesting as a significant interaction between GROUP and VISIT. Age was included as covariate because of the known or potential effects on FA. Additionally, follow-up paired comparisons (t-tests) were used to investigate within group longitudinal changes in FA values.

VBM data of gray matter were analyzed in accordance to the analyses of FA-maps: Both cross-sectional comparisons between ALS patients and controls (two sample t-test) and longitudinal GM changes within both ALS groups were tested (paired t-test and Factorial ANOVA with VISIT as the first factor and GROUP as the second factor).

Differences in total brain volumes between both ALS groups (GM, WM, relative and normalized brain volumes) and to the group of healthy controls were assessed by analysis of covariance, modeling the factor age as co-variable.

### Ethics

The protocol for this trial and supporting CONSORT checklist are available as supporting information; see Checklist S1 and Protocol S1. This study was carried out in strict accordance with the principles expressed in the Declaration of Helsinki. The study was approved by the local ethics committee of the University of Münster and the German Federal Institute for Drugs and Medical Products. All participants in this study gave written informed consent.

## Results

Five placebo-treated patients and 4 ALS patients treated with G-CSF completed the study, including both MRI scans. One patient died of intracranial hemorrhage due to an accidental fall shortly after enrolment and randomization, but before being treated with G-CSF. All individuals who received at least one dose of study medication tolerated the medication well and were able to complete the study.

Clinical tests (total time of JTT, ALSFRS scores, and z-scores of each cognitive domain) did not show a significant difference between both groups in change from baseline to day 30 and day 100 (Table 3). All clinical investigations and MRI scans at the follow-up dates were completed by the ALS patients.

**Table 3:** Primary clinical endpoints during treatment.

	ALS			Controls		
	Baseline	Day 30	Day 100	Baseline	Day 30	Day 100
**ALSFRS scores (mean ± SD)**	35.4**±**7.6	34.6**±**8.5	35.3**±**9.4	35.8**±**7.0	34.1**±**8.9	34.4**±**8.2
**Jebsen Taylor Test [seconds]** * **(mean ± SD)**	49.5**±**7.1	52.1**±**9.2.	50.1**±**6.6	51.7**±**7.7	54.2**±**6.2	50.8**±**8.1
**Cognitive domains** # **(z-scores)**						
Dementia screening	0.005	0.010	−0.007	−0.002	−0.024	0.005
Attention and Working memory	−0.032	−0.008	−0.050	−0.022	−0.046	0.032
Executive function	−0.001	−0.011	−0.052	−0.011	0.041	0.013
Visuospatial skills	−0.081	0.011	0.023	0.045	−0.061	−0.038
Verbal learning and memory	0.027	−0.007	0.018	−0.024	0.019	−0.076

Differences between groups were not significant for any of the time-points, nor were improvement from baseline to mean of Day 30 and Day 100.

*Performance in this test was reflected by total time needed to complete the six subtests; Number of errors was likewise comparable between groups (not significant).

#See table 2 for the detailed neuropsychological test batteries of each cognitive domain.

ALSFRS  =  ALS functional rating scale.

**Figure 2:**
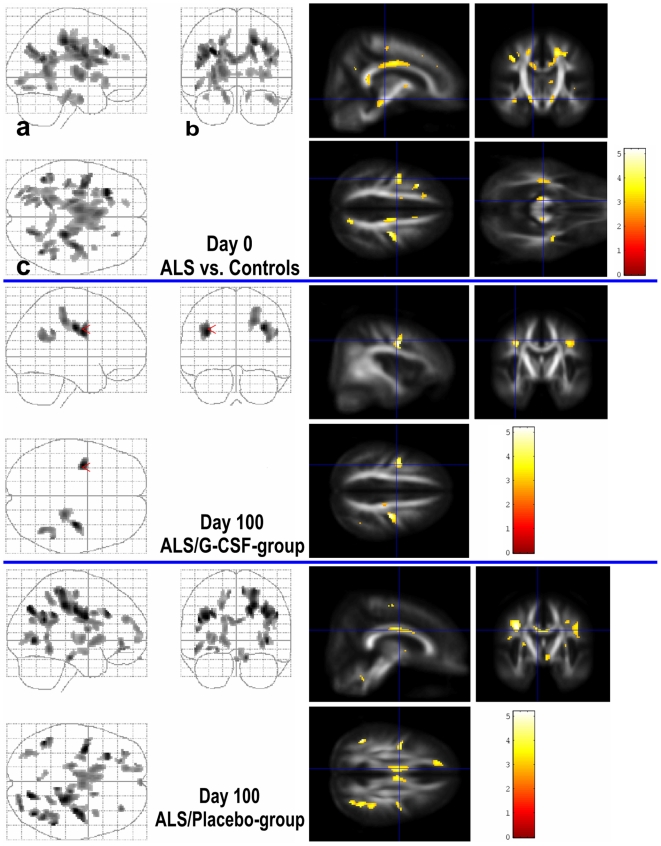
Voxel based analysis of DTI data. SPM “glass brain” representation (left) and slices of voxels (right) with a significant decrease in fractional anisotropy (FA) of patient compared to the healthy controls (ANOVA, *p*<0.001, uncorrected; 50 contiguous voxels). Statistical FA-maps were superimposed on an averaged FA template of the control group. Colored bars represent t-values; display threshold is set at t value >3.16. **Upper row:** Cross-sectional comparison of 10 ALS patients when compared to 32 healthy controls (Visit 1). FA of the ALS patients were significantly reduced in WM areas covering widespread parts of the brain, most prominent in the corticospinal tracts, in subcortical WM of the precentral gyrus, and it's connecting fibres in the corpus callosum. Anatomic pattern of FA changes did not alter significantly when both ALS groups were compared separately to the control group. **Lower and middle row:** Clusters of FA decreases from Visit 1 to Visit 2 in ALS patients treated with G-CSF (middle row) and in the ALS-control group (lower row). ALS patients treated with G-CSF showed small regions of decreased FA, mainly affecting bilateral subcortical WM of the precentral gyrus, whereas the placebo group showed a greater and more widespread decline in FA during the study period. The localization was similar to the clusters of decreased FA in the initial voxel-wise analysis (upper row). Hence, white matter tracts that were initially detected as deficient continued to lose fibre integrity over time.

On cross-sectional comparison, voxel-based analysis revealed a widespread decline in FA in ALS patients when compared to the healthy controls (Figure 2, upper row). These symmetrical WM changes were most prominent in the corticospinal tracts, in subcortical WM of the precentral gyrus, and its connecting fibres in the frontocentral parts of the corpus callosum. Anatomic pattern of FA changes were similar in both ALS groups (at visit 1) were separately compared to the control group.

**Figure 3:**
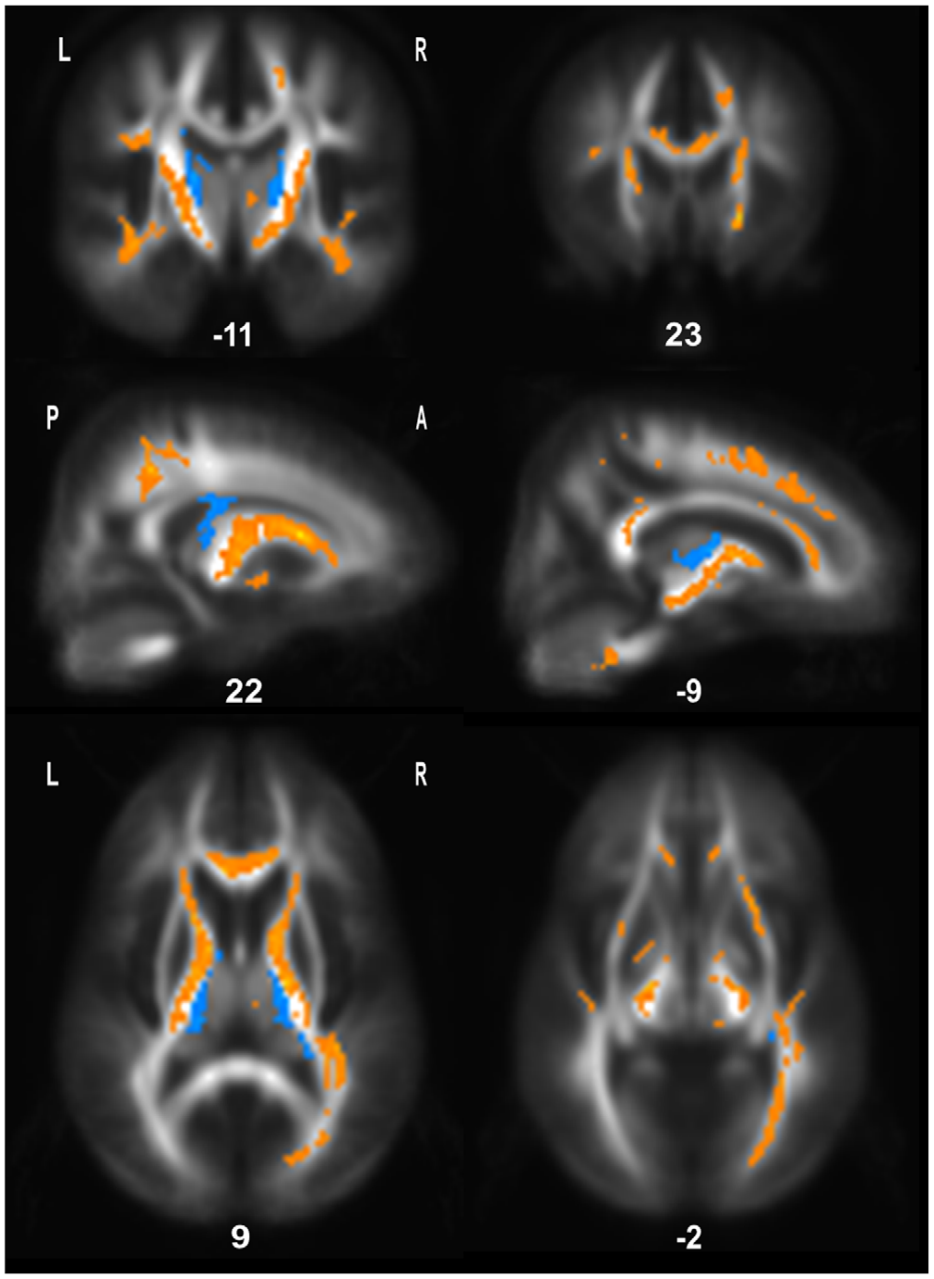
Longitudinal interaction between VISIT and GROUP. Placebo-treated ALS patients showed a greater and more widespread decline in FA over time, compared to ALS patients treated with G-CSF (shown in orange; *p*<0.005, uncorrected; 50 contiguous voxels). These FA changes mainly involved the corticospinal tracts, frontal WM including connecting fibres of the frontal corpus callosum, and temporal WM. Anatomical distribution of decreased FA values in ALS patients treated with G-CSF relative to untreated ALS patients over time are shown in blue (*p*<0.005, uncorrected; 50 contiguous voxels). These clusters were much less widespread, mainly encompassing posterior thalamic regions. Slice positions are indicated in the MNI coordinates.

On longitudinal analysis, ALS patients treated with placebo showed greater and more widespread decline in FA from Visit 1 to Visit 2 (day 100) compared to the ALS patients treated with G-CSF. As shown in Figure 3 the corticospinal tracts (ranging from subcortical regions to the brainstem), frontal WM including connecting fibres of the frontal corpus callosum, and temporal WM showed significantly lower FA values in the untreated ALS group. An interaction in the opposite direction (ALS patients treated with G-CSF showing a greater decline in FA from Visit 1 to Visit 2 compared to ALS-Placebo patients) was also tested. This analysis yielded small symetrical clusters of decreased FA in posterior thalamic regions (Figure 3).

Post hoc t-test of SPM-ANOVA indicated that FA decreased from Visit 1 to Visit 2 mainly bilateral in the subcortical WM of the precentral gyrus in the ALS-G-CSF group (Figure 2, middle row). In the ALS-Placebo group, post hoc analysis also indicated WM changes in these regions. Unlike the G-CSF group the ALS-Placebo patients additionally showed significant clusters of decraesed FA over time in several major white matter tracts including the occipital lobes, frontal regions, and connecting fibres in the corpus callosum (Figure 2, lower row). Interestingly, the localization of FA changes was similar to the clusters of decreased FA in the initial voxel-wise analysis between ALS patients and the healthy controls (Figure 2, upper row). Thus, in particular white matter tracts that were initially detected as deficient in our ALS patients continued to lose fibre integrity over time.

VBM analysis showed no differences in local GM between ALS patients and controls or between both ALS groups. Additionally, no longitudinal GM changes within each ALS group were observed during the study period. There were also no differences in brain volumes between both ALS groups and to the group of healthy controls.

Percentage of patients in each group with at least one AE (mild or moderate in severity) was 75% in the G-CSF, 80% in the placebo group (not significant); no severe AE that led to discontinuation of the study was reported. 72% of AEs in the G-CSF group and 53% in the placebo group were classified as possibly or probably related to the treatment. This effect was also not significant (see Table 4 for details). All treatment related events were within the well-described side effect profile of G-CSF treatment, most often headache (n = 3), bone pain (n = 3), and malaise (n = 2).

**Table 4:** All adverse events (AEs) and treatment-related AEs, listed by system organ class.

Side effects	All Adverse Events		Treatment-related AEs (probable or possible)	
	Placebo	G-CSF	Placebo	G-CSF
**General disorders and administration site conditions**	3	2	1	2
**Nervous system disorders**	5	4	2	2
**Musculoskeletal and connective tissue disorders**	5	3	4	2
**Infections**	1	0	0	0
**Skin and subcutaneous tissue disorders**	0	1	0	1
**Investigations**	1	1	1	1

(n = 11 in the G-CSF group, n = 15 in the placebo group).

Incidences of events were not significantly different between both groups (Fisher's Exact Test; all P's>0.05).

**Figure 4:**
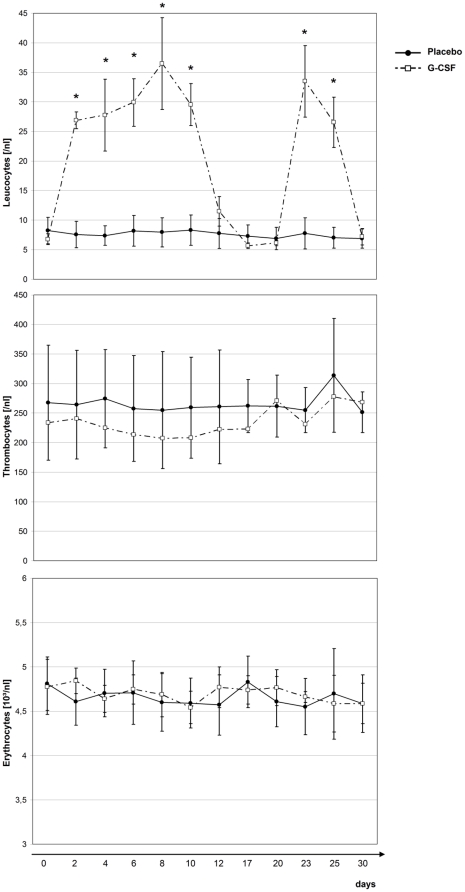
Hematological parameters during the study period. Error bars indicate standard errors of the mean (SEM); * =  significant difference between the G-CSF and placebo group.

Leukozyte count revealed a significant difference between groups for day 2, 4, 6, 8, 10, and 23 and 25 (Figure 4). Highest leukozyte count, reached in one patient on day 8, was 48,390/µl. For platelet count, a reversed pattern was noted, with a slight decrease in the G-CSF group starting on study day 2. However, the difference did not reach statistical significance at any day of the study, with a lowest platelet count of 124/nL on day 8. For erythrocyte count, no significant difference between the study groups was found. Analysis of blood pressure, heart rate, and body temperature revealed no significant difference between groups at any day of the study.

## Discussion

The present study demonstrated feasibility of a subcutaneous treatment of ALS patients with G-CSF over a time course of 25 days, and of the tests and MR measurements conducted. G-CSF was well tolerated. As expected with the very small number of patients, no signals of efficacy could be deducted from the clinical parameters measured (ALSFRS, motor hand functions and neurocognition). Surprisingly, we did detect signals of efficacy using MR imaging: We discovered a reduction in structural disintegration of white matter tracts in ALS patients treated with G-CSF.

So far, only data from one randomized controlled trial with G-CSF in ALS patients are available [14]. In accordance to our findings on imaging, this trial also noted a trend of slowing disease progression following G-CSF treatment. A few other trials demonstrated safety of G-CSF application in ALS patients [15]–[17]. However, this is the first study using advanced neuroimaging techniques as a biomarker in an ALS trial. DTI provides non-invasive information about the integrity of white matter by quantitative measurement of directionality of axonal fibres [18]. It has a proven sensitivity to detect subtle structural brain changes associated with disintegration of WM and, thus, has great diagnostic promise for ALS. Changes in FA have functional relevance since they are correlated to clinical symptoms and histopathological changes in early stages of neurodegenerative diseases [19]. Thus, the slowing of the localized FA decrease that was found in the present study is most likely the structural correlate of a subclinical benefit of G-CSF. This finding is backed by recent studies revealing that DTI has the greatest diagnostic potential for ALS, and has a proven sensitivity to progression of the disease [20]–[22]. Furthermore, in a study of presymptomatic individuals with familial ALS, FA changes were the earliest detectable changes [23]. DTI changes also showed a good correlation with physiological indices and clinical severity in ALS patients [24]–[26].

The FA changes were most prominent in the corticospinal tracts, in subcortical WM of the precentral gyrus, and its connecting fibres in the corpus callosum. This neuroanatomical pattern was in considerable accordance to former studies using voxel-based DTI analysis in ALS patients and reflects the presence of microstructural damage along motor fibres, which was correlated with the degree of motor disability [21], [24], [27]. Degeneration of upper motor neurons usually starts in the primary motor cortex, and secondary degeneration of motor fibres subsequently occurs along the corticospinal tract [28]. However, in the current study VBM did not show significant differences in grey matter between ALS groups or ALS patients and controls, whereas DTI revealed reproducible results, even in this small sample of patients. These findings support former studies concluding that VBM or other volumetric methods might be rather insensitive to image the subtle primary involvement of the motor cortex in neurodegenerative disease, and that DTI is superior to depict an early involvement by imaging the subsequent degeneration of motor fibres [29]–[33].

The extra-motor and widespread FA decline in the untreated patient group during the study period supported the notion, that ALS is a multisystem disorder. These changes in subcortical regions beyond the limits of the primary motor areas are well-known and also clinically relevant [34]–[37]. Post-mortem studies in humans have revealed that lower FA in these regions reflects the extent of astrogliosis and of myelin and, in particular, axonal loss in the white matter [38]. These structural changes have accounted for the slower performance on many motor skills and cognitive tasks [39], [40]. Hence, it has been repeatedly shown, that FA is the most sensitive MR-imaging correlate of executive dysfunction [41], [42], the cognitive domain that is recognized to be particularly affected in ALS patients [43], [44]. Due to the small number of patients, we could not show such correlations. However, although the clinical symptoms that are associated with these FA alterations might be limited, they could have a relevant impact on overall daily function [45]–[48].

In previous experimental studies, G-CSF was shown to protect cultured motoneurons from apoptosis, led to a significant improvement in motor performance, and prolonged overall survival of ALS-mice [5], [6], [49]. Motoneurons in the spinal cord strongly expressed the receptor for G-CSF, and transgenic overexpression of G-CSF in the CNS improved outcome [50]. Parallel to its functions following cerebral ischemia, G-CSF may act as endogenous neuroprotective factor on motoneurons in neurodegenerative diseases. Thus, G-CSF may have a potential as disease-modifying drug in ALS. Furthermore, recent studies have revealed that G-CSF increases microglial recruitment in ALS model mice and restored microglial responses and function [51]. Since inflammation, including microglial dysfunction and T cell infiltration of white matter, is a neuropathological hallmark of ALS [52], G-CSF might also directly improve structural integrity of fiber tracts yia these effects.

The commonly used ALSFRS score is a rather insensitive, non-parametric tool to measure activities of daily living in ALS patients, which could explain some of the negative results [31]. Although in this study the clinical tests were extended by the JTT and a neuropsychological test battery, we also failed to demonstrate a significant clinical benefit of G-CFS treatment, which is due to the small sample size. In recent studies, ALS patients were recruited based on the revised El Escorial criteria resulting in a patient population at an already progressed and relatively late phase of the disease, possibly beyond the therapeutic window. At least 30% of anterior horn neurons are degenerated when patients are recruited at this time of the disease [28]. Establishing an effective treatment at this stage of the disease appears to be very difficult, and indeed recent ALS trials were all negative. Our data demonstrate for the first time widespread and progressive microstructural damage of white matter tracts assessed by FA analysis that can be modulated by drug treatment. Thus, a more intensive training regime in earlier phases of the disease as done here, combined with a drug treatment might be more promising to achieve clinically significant effects in neurodegenerative diseases such as ALS. Future studies should consider a recruitment of patients in earlier stages (probable ALS and probable laboratory supported ALS) in addition to higher doses or longer application of G-CSF, because both experimental and clinical data suggest higher doses of G-CSF to be associated with better functional neurological outcome [5], [6], [53]. For this purpose, sensitive biomarkers in addition to an early therapeutic approach might be necessary. FA analysis of white matter is sensitive to early therapeutic effects, even in a small sample of patients, and thus may represent such an effective marker for therapeutic monitoring in ALS [54].

In conclusion, our results are paving the way for properly powered trials with optimized regimes and escalated G-CSF dosages, combined with voxel-wise DTI analysis as a sensitive tool to quantify subtle brain tissue alterations.

## Supporting Information

Checklist S1 (DOC)

Protocol S1 (DOC)

## References

[1] Mitchell JD, Borasio GD (2007). Amyotrophic lateral sclerosis.. Lancet.

[2] Miller RG, Mitchell JD, Lyon M, Moore DH (2007). Riluzole for amyotrophic lateral sclerosis (ALS)/motor neuron
disease (MND).. Cochrane Database Syst Rev.

[3] Brooks BR (2009). Managing amyotrophic lateral sclerosis: slowing disease
progression and improving patient quality of life.. Ann Neurol.

[4] Zoccolella S, Santamato A, Lamberti P (2009). Current and emerging treatments for amyotrophic lateral
sclerosis.. Neuropsychiatr Dis Treat.

[5] Schabitz WR, Kruger C, Pitzer C, Weber D, Laage R (2008). A neuroprotective function for the hematopoietic protein
granulocyte-macrophage colony stimulating factor (GM-CSF).. J Cereb Blood Flow Metab.

[6] Schneider A, Kruger C, Steigleder T, Weber D, Pitzer C (2005). The hematopoietic factor G-CSF is a neuronal ligand that
counteracts programmed cell death and drives neurogenesis.. J Clin Invest.

[7] Brooks BR, Miller RG, Swash M, Munsat TL (2000). El Escorial revisited: revised criteria for the diagnosis of
amyotrophic lateral sclerosis.. Amyotroph Lateral Scler Other Motor Neuron Disord.

[8] The Amyotrophic Lateral Sclerosis Functional Rating Scale (1996). Assessment of activities of daily living in patients with
amyotrophic lateral sclerosis. The ALS CNTF treatment study (ACTS) phase
I-II Study Group.. Arch Neurol.

[9] Alon G, Sunnerhagen KS, Geurts AC, Ohry A (2003). A home-based, self-administered stimulation program to improve
selected hand functions of chronic stroke.. NeuroRehabilitation.

[10] Lezak MD (2004). Neuropsychological assessment. 4th ed..

[11] Deppe M, Kellinghaus C, Duning T, Moddel G, Mohammadi S (2008). Nerve fiber impairment of anterior thalamocortical circuitry in
juvenile myoclonic epilepsy.. Neurology.

[12] Fein G, Landman B, Tran H, Barakos J, Moon K (2006). Statistical parametric mapping of brain morphology: sensitivity
is dramatically increased by using brain-extracted images as
inputs.. Neuroimage.

[13] Smith SM, Zhang Y, Jenkinson M, Chen J, Matthews PM (2002). Accurate, robust, and automated longitudinal and cross-sectional
brain change analysis.. Neuroimage.

[14] Nefussy B, Artamonov I, Deutsch V, Naparstek E, Nagler A (2009). Recombinant human granulocyte-colony stimulating factor
administration for treating amyotrophic lateral sclerosis: A pilot
study.. Amyotroph Lateral Scler.

[15] Cashman N, Tan LY, Krieger C, Madler B, Mackay A (2008). Pilot study of granulocyte colony stimulating factor
(G-CSF)-mobilized peripheral blood stem cells in amyotrophic lateral
sclerosis (ALS).. Muscle Nerve.

[16] Tarella C, Rotella S, Gualandi F, Melazzini M, Scime R (2009). Consistent bone marrow-derived cell mobilization following
repeated short courses of granulocyte-colony-stimulating factor in patients
with amyotrophic lateral sclerosis: results from a multicenter prospective
trial.. Cytotherapy.

[17] Zhang Y, Wang L, Fu Y, Song H, Zhao H (2008). Preliminary investigation of effect of granulocyte colony
stimulating factor on amyotrophic lateral sclerosis.. Amyotroph Lateral Scler.

[18] Le Bihan D, Mangin JF, Poupon C, Clark CA, Pappata S (2001). Diffusion tensor imaging: concepts and
applications.. J Magn Reson Imaging.

[19] Gouw AA, Seewann A, Vrenken H, van der Flier WM, Rozemuller JM (2008). Heterogeneity of white matter hyperintensities in
Alzheimer's disease: post-mortem quantitative MRI and
neuropathology.. Brain.

[20] Agosta F, Pagani E, Petrolini M, Caputo D, Perini M (2010). Assessment of White Matter Tract Damage in Patients with
Amyotrophic Lateral Sclerosis: A Diffusion Tensor MR Imaging Tractography
Study.. AJNR Am J Neuroradiol.

[21] Dengler R, von Neuhoff N, Bufler J, Krampfl K, Peschel T (2005). Amyotrophic lateral sclerosis: new developments in diagnostic
markers.. Neurodegener Dis.

[22] Agosta F, Pagani E, Petrolini M, Sormani MP, Caputo D (2010). MRI predictors of long-term evolution in amyotrophic lateral
sclerosis.. Eur J Neurosci.

[23] Ng MC, Ho JT, Ho SL, Lee R, Li G (2008). Abnormal diffusion tensor in nonsymptomatic familial amyotrophic
lateral sclerosis with a causative superoxide dismutase 1
mutation.. J Magn Reson Imaging.

[24] Grosskreutz J, Peschel T, Unrath A, Dengler R, Ludolph AC (2008). Whole brain-based computerized neuroimaging in ALS and other
motor neuron disorders.. Amyotroph Lateral Scler.

[25] Iwata NK (2007). [Objective markers for upper motor neuron involvement in
amyotrophic lateral sclerosis].. Brain Nerve.

[26] Pyra T, Hui B, Hanstock C, Concha L, Wong JC (2010). Combined structural and neurochemical evaluation of the
corticospinal tract in amyotrophic lateral sclerosis.. Amyotroph Lateral Scler.

[27] Stanton BR, Shinhmar D, Turner MR, Williams VC, Williams SC (2009). Diffusion tensor imaging in sporadic and familial (D90A SOD1)
forms of amyotrophic lateral sclerosis.. Arch Neurol.

[28] Swash M, Ingram D (1988). Preclinical and subclinical events in motor neuron
disease.. J Neurol Neurosurg Psychiatry.

[29] Canu E, McLaren DG, Fitzgerald ME, Bendlin BB, Zoccatelli G (2009). Microstructural Diffusion Changes are Independent of
Macrostructural Volume Loss in Moderate to Severe Alzheimer's
Disease.. J Alzheimers Dis.

[30] Duning T, Warnecke T, Mohammadi S, Lohmann H, Schiffbauer H (2009). Pattern and progression of white-matter changes in a case of
posterior cortical atrophy using diffusion tensor imaging.. J Neurol Neurosurg Psychiatry.

[31] Turner MR, Kiernan MC, Leigh PN, Talbot K (2009). Biomarkers in amyotrophic lateral sclerosis.. Lancet Neurol.

[32] Muller MJ, Greverus D, Weibrich C, Dellani PR, Scheurich A (2007). Diagnostic utility of hippocampal size and mean diffusivity in
amnestic MCI.. Neurobiol Aging.

[33] Sach M, Winkler G, Glauche V, Liepert J, Heimbach B (2004). Diffusion tensor MRI of early upper motor neuron involvement in
amyotrophic lateral sclerosis.. Brain.

[34] Abrahams S, Goldstein LH, Suckling J, Ng V, Simmons A (2005). Frontotemporal white matter changes in amyotrophic lateral
sclerosis.. J Neurol.

[35] Mezzapesa DM, Ceccarelli A, Dicuonzo F, Carella A, De Caro MF (2007). Whole-brain and regional brain atrophy in amyotrophic lateral
sclerosis.. AJNR Am J Neuroradiol.

[36] Senda J, Ito M, Watanabe H, Atsuta N, Kawai Y (2009). Correlation between pyramidal tract degeneration and widespread
white matter involvement in amyotrophic lateral sclerosis: a study with
tractography and diffusion-tensor imaging.. Amyotroph Lateral Scler.

[37] van der Graaff MM, de Jong JM, Baas F, de Visser M (2009). Upper motor neuron and extra-motor neuron involvement in
amyotrophic lateral sclerosis: a clinical and brain imaging
review.. Neuromuscul Disord.

[38] Gouw AA, Seewann A, Vrenken H, van der Flier WM, Rozemuller JM (2008). Heterogeneity of white matter hyperintensities in
Alzheimer's disease: post-mortem quantitative MRI and
neuropathology.. Brain.

[39] Kim SH, Park JS, Ahn HJ, Seo SW, Lee JM (2010). Voxel-Based Analysis of Diffusion Tensor Imaging in Patients with
Subcortical Vascular Cognitive Impairment: Correlates with Cognitive and
Motor Deficits.. J Neuroimaging.

[40] Mascalchi M, Filippi M, Floris R, Fonda C, Gasparotti R (2005). Diffusion-weighted MR of the brain: methodology and clinical
application.. Radiol Med.

[41] Nitkunan A, Barrick TR, Charlton RA, Clark CA, Markus HS (2008). Multimodal MRI in cerebral small vessel disease: its relationship
with cognition and sensitivity to change over time.. Stroke.

[42] O'Sullivan M, Morris RG, Huckstep B, Jones DK, Williams SC (2004). Diffusion tensor MRI correlates with executive dysfunction in
patients with ischaemic leukoaraiosis.. J Neurol Neurosurg Psychiatry.

[43] Giordana MT, Ferrero P, Grifoni S, Pellerino A, Naldi A (2010). Dementia and cognitive impairment in amyotrophic lateral
sclerosis: a review.. Neurol Sci.

[44] Phukan J, Pender NP, Hardiman O (2007). Cognitive impairment in amyotrophic lateral
sclerosis.. Lancet Neurol.

[45] Kavcic V, Ni H, Zhu T, Zhong J, Duffy CJ (2008). White matter integrity linked to functional impairments in aging
and early Alzheimer's disease.. Alzheimers Dement.

[46] Charlton RA, Barrick TR, McIntyre DJ, Shen Y, O'Sullivan M (2006). White matter damage on diffusion tensor imaging correlates with
age-related cognitive decline.. Neurology.

[47] Charlton RA, Schiavone F, Barrick TR, Morris RG, Markus HS (2010). Diffusion tensor imaging detects age related white matter change
over a 2 year follow-up which is associated with working memory
decline.. J Neurol Neurosurg Psychiatry.

[48] Schiavone F, Charlton RA, Barrick TR, Morris RG, Markus HS (2009). Imaging age-related cognitive decline: A comparison of diffusion
tensor and magnetization transfer MRI.. J Magn Reson Imaging.

[49] Pitzer C, Kruger C, Plaas C, Kirsch F, Dittgen T (2008). Granulocyte-colony stimulating factor improves outcome in a mouse
model of amyotrophic lateral sclerosis.. Brain.

[50] Tanaka M, Kikuchi H, Ishizu T, Minohara M, Osoegawa M (2006). Intrathecal upregulation of granulocyte colony stimulating factor
and its neuroprotective actions on motor neurons in amyotrophic lateral
sclerosis.. J Neuropathol Exp Neurol.

[51] Yamasaki R, Tanaka M, Fukunaga M, Tateishi T, Kikuchi H (2010). Restoration of microglial function by granulocyte-colony
stimulating factor in ALS model mice.. J Neuroimmunol.

[52] Henkel JS, Beers DR, Zhao W, Appel SH (2009). Microglia in ALS: the good, the bad, and the
resting.. J Neuroimmune Pharmacol.

[53] Minnerup J, Heidrich J, Wellmann J, Rogalewski A, Schneider A (2008). Meta-analysis of the efficacy of granulocyte-colony stimulating
factor in animal models of focal cerebral ischemia.. Stroke.

[54] Filippi M, Agosta F, Abrahams S, Fazekas F, Grosskreutz J (2010). EFNS guidelines on the use of neuroimaging in the management of
motor neuron diseases.. Eur J Neurol.

