# Supplementary material for: G-CSF Prevents the Progression of Structural Disintegration of White Matter Tracts in Amyotrophic Lateral Sclerosis: A Pilot Trial
Source: PLoS One. 2011 Mar 14;6(3):e17770. doi: 10.1371/journal.pone.0017770 (PMC3056779; doi:10.1371/journal.pone.0017770)
Supplement: Protocol S1 — (DOC) [file pone.0017770.s002.doc]

**G-CSF in the treatment of ALS**

Prof. Wolf-Rüdiger Schäbitz, MD

Dr. Thomas Duning, MD

Dr. Tobias Warnecke, MD

University Hospital of Münster, Germany

**1. Aim of the study and Introduction**

- *Why the study is conducted?*
The long term goal of the study is the pharmacological treatment of neurodegenerative diseases (including Alzheimer's dementia, vascular dementia, Parkinson's disease and amyotrophic lateral sclerosis) by drugs that stimulate neurogenesis in the central nervous system (CNS) and have neuroprotective effects.

The immediate objective of the study is to demonstrate that systemically applied haematopoietic growth factors increase neurogenesis and lead to a neuroprotection in amyotrophic lateral sclerosis (ALS) patients, which cause an improvement of motor functions and cognitive .
 *-What is known?*
Various animal experiments in recent years have shown that the adult brain is able to produce new nerve cells (neurogenesis). The old hypothesis that the formation of new nerve cells at birth is complete was proved to be obsolete [Kempermann et al. 1998].

Neurogenesis particularly takes place in the subventricular zone and dentate gyrus of the hippocampus, a brain region which is important for learning and remembering. Every day several thousands of so-called "granule -cells" in the hippocampus of mammals are evolved [Roy et al. 2000, Jin et al. 2003, Nunes et al. 2003, Kempermann et al. 2004].

The neurogenesis in the adult brain is modulated by various environmental factors, such as physical activity, mental stress or learning [Nilsson et al. In 1999, Van Praag et al. 1999 Döbrössy et al. 2003, Drapeau et al. 2003 Dragansky et al. 2004]. Neurogenesis in the adult brain can also be stimulated by subcutaneous or intravenous administration of pharmacological substances. Animal studies have shown that systemic administration of various peripheral growth factors (Basic Fibroblast Growth Factor, Insulin-like growth factor-I) leads to an increased neurogenesis in the subventricular zone and dentate gyrus of adult rats [Wagner et al. 1999, Aberg et al. 2000].

Hemato-and granulopoietic growth factors, such as erythropoietin (EPO) and granulocyte - colony stimulating factor (G-CSF) do not only control the proliferation and differentiation of progenitor cells of hematopoiesis, but also have a neuroprotective and neurotrophic effect. Several animal studies have shown that there were different effects of EPO and G-CSF on neurons. These effects are mainly mediated by specific receptors (EPOR and G-CSFR). Therefore, it is postulated that the brain might have a specific, local EPO / EPOR and G-CSF/G-CSFR – system [Dame et al. 2001, Sirén et al. 2001 Schäbitz et al. 2003, Ghezzi et al. 2004, et al Jelkmenn. 2004, Maiese et al. 2005 Schäbitz et al. (Unpublished results)]. Since G-CSF can cross the blood-brain barrier, a direct effect of systemically administered G-CSF to the cerebral G-CSF receptor is possible, [Schäbitz et al. (Unpublished results)]. Experimental studies have demonstrated that treatment with G-CSF in mice or rats with brain ischemia can improve motor function [Schäbitz et al. 2003, Shyu et al. 2004, Gibson et al. 2005 Schäbitz et al. (Unpublished results)]. Even mice with an autoimmune encephalomyelitis have benefited from a treatment with growth factors [Zhang et al. 2005].

The expression of growth factors in the cortex and in the hippocampus decreases with age [Chung et al. 2004]. In animal experiments we could demonstrate that G-CSF stimulates neurogenesis in the dentate gyrus of adult rats, which points to a promoting effect of neurogenesis in the adult brain [Wang et al. 2004].
The influence of neurogenesis in the adult hippocampus to memory functions is not yet clear. However, it was recently demonstrated that newly generated neurons in the adult mammalian brain could mature to functioning neurons [Van Praag et al. 2002].

Amyotrophic lateral sclerosis (ALS) is a devastating neurodegenerative disease that results in progressive loss of motoneurons, motor weakness and death within 1 to 5 years after disease onset. Meanwhile it is known that the neurodegenerative changes in ALS are not limited to motor neurons. ALS patients also suffer from cognitive impairment in multiple domains, including memory dysfunction. The incidence (incidence rate per year) of ALS is 1.2 to 1.8 / 100000 and is much higher than previously assumed [Strong et al. 2003]. Therapeutic options remain limited despite a substantial number of approaches that have been tested clinically. Since the exact disease-causing mechanism(s) is unclear at present, a rational approach is to give general trophic support to motoneurons, e.g. by growth factors. Various neurotrophic factors have been investigated. Failure in these trials has been largely ascribed to problems of insufficient dosing or inability to cross the blood-brain barrier (BBB). Unpublished data have recently shown that treatment with G-CSF could improve motor function and prolonged survival in an animal model of ALS.

*- Prior studies?*Several animal studies exist that have shown the neuroprotective and neurotrophic effects of G-CSF [Schäbitz et al. 2003]. In these studies systemically applicated G-CSF - similar to EPO – prevented the programmed cell death (apoptosis counteracts) of nerve cells, and thus demonstrated a neuroprotective effect. Furthermore, it was shown that the expression of G-CSF/G-CSFR increased after G-CSF application in ischemic brain tissue (especially in the penumbra). Treatment with G-CSF has resulted in a reduction of infarct volume and improved clinical outcome (recovery of sensory-motor functions). Interestingly, in the preliminary animal studies have also demonstrated that the administration of G-CSF do not only increase neurogenesis in the dentate gyrus of experimental animals suffering from a stroke, but also in all healthy animals.

*-What should be investigated?*
This study is designed as a pilot study to evaluate whether the systemic subcutaneous application of G-CSF could positively affect motor skills and neurocognitive deficits in patients with ALS and whether actual structural cerebral changes could be detected by diffusion weighted MRI.

**2. Experimental design and methods**

To assess motor, the Jebsen Taylor Test is conducted. The Jebsen-Taylor Test (JTT) is a frequently applied clinical and research test for the validation of hand functions that are needed in everyday life [Jebsen et al. 1969, Hackel et al. 1992]. The test examines both the dominant as well as the non-dominant hand with a series of seven subtests related to activities of daily living.

The neuropsychological test battery was designed to assess a full range of cognitive functions and consists of routinely used standardised neuropsychological tests. A detailed description may be found in Lezak et al [Lezak 2004]. Single tests were grouped into three z-transformed composite scores by principal component analysis using an oblique (Oblimin with Kaiser-normalisation) rotation and including coefficients with absolute values above 0.35. The composite scores reflect the cognitive domains of verbal memory, word fluency, and executive functions.

To determine the influence of G-CSF on the central nervous system, MRI scans will be obtained at two time points. The first scan will be done on day 0 (before administration of study medication), the second scan will be obtained on day 100. The patients are examined in a 3-Tesla magnetic resonance imaging (MRI) scanner. High-resolution structural image data of the whole brain will be obtained. In addition, diffusion tensor images were acquired to cover possible microstructural cerebral changes during the study period. The total scanning time is about 35-50 minutes.

In detail, MR-Image data will be obtained on a 3.0 T system with a high resolution structural T1-weighted 3D turbo-field-echo sequence (reconstructed after zero filling to 512x410x320 cubic voxels, edge length 0.5 mm), as well as T2-weighted, and FLAIR imaging. For DTI we employ echo planar imaging (EPI) with 20 diffusion directions (36 slices, thickness 3.6 mm, matrix 128 x 128, inplane resolution 1.8 x 1.8 mm).

MRI examinations were performed at Day 0 and Day 100 of the study.

Furthermore, blood pressure, body temperature measurements and blood samples (blood count, differential blood count, and immune status) are carried out. An increase in absolute white blood cell count to a value> 70000/μl, a hematocrit> 48% or an absolute decrease in platelet count to a value less than <50000/μl will lead to a withdrawal from the study. If the absolute white blood cell count rise to a value greater 50000/μl, or hemoglobin increases more than 2 g / dL or absolute platelet count props to a value less 100000/μl within a few days, daily blood samples are carried out. If necessary, the dose of G-CSF will be adjusted (e. g. halving the dose to 5μg/kg/day).

The study design is shown in Table 1.

**G-CSF in the treatment of ALS**

**Overall n = 40 participants**

**- group I G-CSF application in 20 ALS patients**

**- group II Placebo application in 20 ALS patients**

**Randomized allocation into three subgroups, each with n = 20**:

- Granulocyte – Colony Stimulating Factor (G-CSF) 10 µg/kg/Tag
- Placebo (saline solution) 0,1 ml/kg/Tag

Day 0 Structured Interview (1 hour)

ALS functional rating scale (ALSFRS)

Neuropsychological testing (2 hours)

Jebsen-Taylor Test (15 min)

Blood pressure and body temperature

Blood collection

MRI

Day 1 - 10 Blood pressure and body temperature

s. c. application of G-CSF/Placebo

Day 2 Blood collection

Day 4 Blood collection

Day 6 Blood collection

Day 8 Blood collection

Day 10 Blood collection

Day 12 Blood collection, Blood pressure and body temperature

Day 17 Blood collection, Blood pressure and body temperature

Day 20 - 25 Blood pressure and body temperature

s. c. application of G-CSF/Placebo

Day 20 Blood collection

Day 23 Blood collection

Day 25 Blood collection

Day 30 Blood collection

Blood pressure and body temperature

ALS functional rating scale (ALSFRS)

Neuropsychological testing (2 hours)

Jebsen-Taylor Test (15 min)

Day 40 Blood collection

Blood pressure and body temperature

Day 60 Blood collection

Blood pressure and body temperature

Day 80 Blood collection

Blood pressure and body temperature

Day 100 ALS functional rating scale (ALSFRS)

Neuropsychological testing (2 hours)

Jebsen-Taylor Test (15 min)

Blood pressure and body temperature

Blood collection

MRI

*- What is the design of the study? Is it*

*- A pharmacological, clinical pharmacology, clinical or other biomedical study?*Clinical-pharmacological (administration of growth factors) and biomedical (measurement of motor function, MRI and cognition) study.

- *An open or single-or double-blind study?*
It is a placebo-controlled, double-blind study.

*- Randomized study?*The allocation of subjects to the different test substances is randomized.

*- Diagnostic, therapeutic, compatibility or exclusive scientific study?*It is a scientific and therapeutic study.

*- By whom will the participants be medically supervised before, during and after study? (Agreement with the GP, control of other medications?)*The supervision of the study participants is made by the investigator, Dr. Thomas Duning, medical resident at the Department of Neurology, University Hospital of Münster (UKM).

*- Duration of the study*The study lasts one to three years.

*- Will interim results be analyzed to detect a trend? What criteria and which consequences are drawn for the participants?*Because of the double-blind nature of the study no interim results can be analyzed.

- *What kind of documentation is provided?*

Clinical data will be stored as data files. For each subject a test code will be assigned. The assignment list of drugs and codes is maintained by the Pharmacy of the University Hospital of Münster.

*- Is the involvement of a statistician provided and what statistical methods will be used?*For the major analysis of the data an analysis of variance (ANOVA) with the factors substance group (G-CSF or placebo) and study day will be conducted. If significant group differences appear, further tests using post hoc- analyses will be conducted.

- *Will the medical confidentiality and privacy provisions be respected?*
The medical confidentiality and privacy provisions are respected.

*- Will the trial participants get a fee (expenses paid, etc.)? How much?*
No.

*- Will the participating investigator get a fee? How much?*
No.

- *Number of participants (in case of comparative studies please name the number of patients in each groups sharing)*A total of up to 40 subjects should be included, 20 in each of the two groups.

- *Age and Gender of participants (please indicate the age of the participant and the upper and lower limits of the exclusion criteria)*Only subjects who suffer from amyotrophic lateral sclerosis will be included. There is not an age limit.

*- Status: Are the participants
 a) healthy persons?* No.

 *b) pregnant or breastfeeding women?* No.
 *c) relevant sufferers? (Please specify the disease and the state)*
 Patients with definite amyotrophic lateral sclerosis.

 *d) Inpatient or outpatient?* Outpatients

 *e) Persons suffering from other diseases? (In particular mental illness, and are there doubts about the business or legally competence?)* No

*- What are other inclusion criteria (for example: allowed concomitant medications)?*No further inclusion criteria.

- *What other exclusion criteria (such as advanced kidney or liver failure, etc. prohibited concomitant medications) are provided?*Subjects with a WBC> 10000/μl (normal range: 4000-10000/μl be), a hematocrit> 48% or a platelet count <150000/μl (normal range: 150000-450000/μl) at initial blood tests will be excluded. Furthermore, a known allergy to substances used in this study, a congenital neutropenia, an alcohol dependence, and in women of childbearing age, a positive pregnancy test will lead to exclusion. Conditions leading to exclusion are severe rheumatoid arthritis, severe liver and kidney disease, recurrent venous thrombosis, recurrent pulmonary embolism, a refractory arterial hypertension, severe blood disease and myocardial infarction.
*- The risk-benefit ratio: What benefits are expected from the results of the study*

 *a) for the participants?* The trial participants may benefit from a possible improvement of the cognitive and motor deficits.

 *b) for medicine?* The long-term benefit is to improve the pharmacological treatment of neurodegenerative diseases with limited therapeutic options like ALS.

c) *for science?* The direct benefit for science is a betterunderstanding of the function of neurogenesis and neuroprotection in the adult human brain and its pharmacological interference. The results could likely transferred to other neurodegenerative disease with cognitive and motor deficits.

*- Risks of the study*: *What kind of risks?*Common side effects (> 10%) of G-CSF are headache, musculoskeletal pain, fatigue, leukocytosis and thrombocytopenia. Both head and bone pain can be successfully treated with paracetamol. Less frequent (<10%) side effects that might occur are elevated alkaline phosphatase and increased lactate dehydrogenase. Rare (<1%) side effects are allergic reactions, a ruptured spleen, increased SGOT, hyperuricaemia or worsening of rheumatoid arthritis. The side effects are related to the administered dose and the time of application.

*- Have criteria been defined which lead to an interruption of the whole trial? If so, what criteria?*

The study will be terminated as soon as indicated by the occurrence of serious adverse events described above. These include in particular a splenic rupture, thromboembolic events and severe allergic reactions.
 *- How are the trial participants be informed about the nature, significance and scope of the study?*
The trial participants are informed about the nature, significance and scope of the study in person and on the basis of standardized consent forms by the investigator.

*- How will the trial participants declare their consent to participate in the study?*The consent will be carried out by signature on the patient's consent form.

*- What kind of drugs are tested? (Please include the following: name, description, chemistry of the substance, pharmacology, toxicology, pharmacokinetics)*The Granulocyte - Colony Stimulating Factor (G-CSF) is tested. It is a naturally occurring glycoprotein that is produced artificially by recombinant DNA technology. There is a glycosylated (Lenograstim) and a non-glycosylated (filgrastim) form. Lenograstim is produced in ovary cells from Chinese hamsters and Filagrastim in the bacterium Escherichia coli. In our study Filgrastim (r-= metHuG-CSF) is used. The clearance of filgrastim follows a first-order kinetics. The half-life in serum is about 3-4h with a clearance of about 0.6 ml per minute and kg. The maximum serum concentration after subcutaneous administration is achieved after 2-8h. In healthy blood donors a dose of 10 or 2x5 g / kg KG / day is given to mobilize peripheral blood stem cells from bone marrow. The “peak number" of the CD34 + - cells can be detected 5 days after leukapheresis.

A saline solution will be used as placebo substance.

- *Is the used substance approved by the Federal Institute for Drugs and Medical Devices?*
The test substance is already used as a drug for different indications. It is also used in healthy people.

**References**

Aberg M, Aberg D, Hedbäcker H, Oscarsson J, Eriksson P. Peripheral Infusion of IGF-I Selectively Induces Neurogenesis in the Adult Rat Hippocampus. J. Neurosci. 2000, 20(8): 2896-2903.

Chung YH, Kim SI, Joo KM, Kim YS, Lee WB, Yun KW, Cha CI. Age-related changes in erythropoetin immunoreactivity in the cerebral cortex and hippocampus of rats. Brain Research 2004, 1018. 141-146.

Dame C, Juul SE, Christensen RD. The Biology of Erythropoetin in the Central Nervous System and Its Neurotrophic and Neuroprotective Potential. Biol Neonate 2001, 79: 228-235.

Döbrössy MD, Drapeau E, Aurousseau C, Moal ML, Piazza PV, Abrous DN. Differential effects of learning on neurogenesis: learning increases or decreases the number of newly born cells depending on their birth date. Molecular Psychiatry 2003, 8: 974-982.

Drapeau E, Mayo W, Aurousseau C, Moal ML, Piazza P-V, Abrous DN. Spatial memory performances of aged rats in the water maze predict levels of hippocampal neurogenesis. PNAS 2003, 100 (24): 14385-14390.

Ghezzi P, Brines M. Erythropoetin as an antiapoptotic, tissue-protective cytokine. Cell Death and Differentiation 2004, 11: 37-44.

Gibson CL, Bath PM, Murphy SP. G-CSF reduces infarct volume and improves functional outcome after transient focal cerebral ischemia in mice. J Cereb Blood Flow Metab. 2005, 19: [Epub ahead of print]

Hackel ME, Wolfe GA, Bang SM, Canfield JS. Changes in hand function in the aging adult as determined by the Jebsen Test of Hand Function. Phys Ther. 1992, 72(5): 373-7.

Jebsen RH, Taylor N, Trieschmann RB, Trotter MJ, Howard LA. An objective and standardized test of hand function. Arch Phys Med Rehabil 50(6): 311-9

Jelkmann W, Wagner K. Benefical and ominous aspects of the pleotropic action of erythropoetin. Annals of Hematology 2004

Jin K, Sun Y, Xie L, Peel A, Mao XO, Batteur S, Greenberg DA. Directed migration of neuronal precursors into the ischemic cerebral cortex and striatum. Molecular and Cellular Neuroscience 2003, 24: 171-189.

Kempermann G, Gage FH. Closer to neurogenesis in adult humans. Nature Medicine 1998, 4(5): 555-557.

Kempermann G, Wiskott L, Gage FH. Functional significance of adult neurogenesis. Current Opinion in Neurobiology 2004, 14: 186-191.

Lezak MD Neuropsychological assessment. New York, Oxford: Oxford University Press 2004.

Maiese K, Li F, Zhong Chong Z. New Avenues of Exploration for Erythropoetin. JAMA 2005, 293: 90-95.

Nilsson M, Perfilieva E, Johansson U, Orwar O, Eriksson PS. Enriched Environment Increases Neurogenesis in the Adult Rat Dentate Gyrus and Improves Spatial Memory. J Neurobiol. 1999, 39(4): 569-578.

Nunes MC, Roy NS, Keyoung HM, Goodman RR, McKhann G, Jiang L, Nedergaard M, Goldmann SA. Identification and isolation of multipotential neural progenitor cells from the subcortical white matter of the adult human brain. Nature Medicine 2003, 9(4): 439-447.

Roy NS, Wang S, Jiang L, Benraiss A, Harrison-Restelli C, Fraser R, Couldwell WT, Kawaguchi A, Okano H, Nedergaard M, Goldmann SA. In vitro neurogenesis by progenitor cells isolated from the adult human hippocampus. Nature Medicine 2000, 6(3). 271-277.

Schäbitz WR, Kollmar R, Schwaninger M, Juettler E, Bardutzky J, Schölzke MN, Sommer C, Schwab S. Neuroprotective Effect of Granulocyte Colony-Stimulating Factor After Focal Cerebral Ischemia. Stroke 2003, 34: 745-751.

Shyu W-C, Lin S-Z, Yang H-I, Tzeng Y-S, Pang C-H, Yen P-S, Li H. Functional Recovery of Stroke Rats Induced by Granulocyte Colony-Stimulating Factor – Stimulated Stem Cells. Circulation 2004, 110: 1847-1854.

Sirén AL, Ehrenreich H. Erythropoetin – a novel concept for neuroprotection. Eur Arch Psychiatry Clin Neurosci 2001, 251: 179-184.

Strong M, Rosenfeld J. Amyotrophic lateral sclerosis: a review of current concepts. Amyotroph Lateral Scler Other Motor Neuron Disord. 2003, 4(3): 136-143.

Van Praag H, Christie BR, Sejnowski TJ, Gage FH. Running enhances neurogenesis, learning and long-term potentation in mice. Neurobiology 1999, 96(23): 13427-13431.

Van Praag H, Schinder AF, Christie BR, Toni N, Palmer TD, Gage FH. Functional neurogenesis in the adult hippocampus. Nature 2002, Vol 415: 1030-1034.

Wagner JP, Black IB, Bloom ED. Stimulation of Neonatal and Adult Brain Neurogenesis by Subcutaneous Injection of Basic Fibroblast Growth Factor. J. Neurosci. 1999, 19(14): 6006-6016.

Wang L, Zhang Z, Wang Y, Zhang R, Chopp M. Treatment of Stroke With Erythropoetin Enhances Neurogenesis and Angiogenesis and Improves Neurological Function in Rats. Stroke 2004, 35: 1732-1737.

Zhang J, Li Y, Cui Y, Chen J, Lu M, Elias SB, Chopp M. Erythropoetin treatment improves neurological functional recovery in EAE mice. Brain Research 2005, 1034: 34-39.
